# Natural Killer Cells Adapt to Cytomegalovirus Along a Functionally Static Phenotypic Spectrum in Human Immunodeficiency Virus Infection

**DOI:** 10.3389/fimmu.2018.02494

**Published:** 2018-11-12

**Authors:** Kayla A. Holder, Julie Lajoie, Michael D. Grant

**Affiliations:** ^1^Immunology and Infectious Diseases Program, Division of BioMedical Sciences, Faculty of Medicine, Memorial University of Newfoundland, St. John's, NL, Canada; ^2^Department of Medical Microbiology, University of Manitoba, Winnipeg, MB, Canada

**Keywords:** HCMV, NK, HIV, CD16, NKG2C, CD57, FcεRIγ, Tim-3

## Abstract

Events related to HCMV infection drive accumulation of functionally enhanced CD57^pos^NKG2C^pos^ adapted NK cells. We investigated NK cell adaptation to HCMV along a proposed continuum progressing from acute activation through maturation and memory formation towards functional exhaustion. Acute exposure to conditioned medium collected 24 h after HCMV infection (HCMVsn) increased NK cell cytotoxicity for all HCMV-seronegative and seropositive donors tested, with mean 38 and 29% boosts in natural and antibody-dependent cell-mediated cytotoxicity (ADCC), respectively. Increases in NK cell cytotoxicity were completely abrogated by blocking type I interferon (IFN) receptors and equivalent responses occurred with exposure to IFN-α2 alone at the same concentration present in HCMVsn. To study longer term effects of HCMV infection, we focused on three groups of human immunodeficiency virus (HIV)-infected subjects distinguished as HCMV-seronegative or HCMV-seropositive with either high (>20%) or low (<6%) fractions of their NK cells expressing NKG2C. The NK cells of all three HIV-infected groups responded to HCMVsn and IFN-α2 in a manner similar to the NK cells of either HCMV-seronegative or seropositive controls. Neither HCMV status, nor the extent of phenotypic evidence of adaptation to HCMV infection significantly affected mean levels of ADCC or CD16-mediated NK cell degranulation and IFN-γ production compared between the HIV-infected groups. Levels of IFN-γ production correlated significantly with the fraction of NK cells lacking FcεRIγ (FcRγ), but not with the fraction of NK cells expressing NKG2C. There was negligible expression of exhaustion markers Lag-3 and PD-1 on NK cells in any of the groups and no significant difference between groups in the fraction of NK cells expressing Tim-3. The fraction of NK cells expressing Tim-3 was unaffected by CD16 stimulation. Relative to the total NK cell population, responses of Tim-3-expressing cells to CD16 stimulation were variably compromised in HCMV seronegative and seropositive groups. In general, NK cell function in response to signaling through CD16 was well preserved in HIV infection and although HCMV had a clear effect on NK cell FcRγ and NKG2C expression, there was little evidence that the level of adaptation to HCMV infection affected CD16-dependent NK cell signaling in HIV infection.

## Introduction

Natural killer (NK) cells provide defense against malignancy and infection by recognizing certain alterations in affected cells and responding accordingly. Activation of NK cells against altered host cells is regulated by integration of positive and negative signals received through members of a relatively small set of germ-line encoded receptors. While NK cells recognize altered host cells and mediate effector functions without prior exposure to the altered cells, NK cell functional competence depends upon previous selective engagement of an inhibitory NK cell receptor with a class I human histocompatibility-linked antigen (HLA) (1). This developmental education process enables NK cells to mediate cytotoxicity and/or produce cytokines when encountering altered host cells, while ensuring appropriate levels of self-tolerance (2–5). It also illustrates that NK cell function is not constitutive upon lineage determination, but dependent upon subsequent relay of specific signals. Selective NK cell education at this developmental stage raises the possibility of further maturation within select subsets under conditions associated with infections or malignancy. Such NK cell maturation clearly takes place in murine cytomegalovirus (MCMV) infection. In this setting, cytokine production and specific receptor ligand interactions between Ly49H and MCMV m157 drive a subset of NK cells expressing the Ly49H activating receptor to selectively expand, persist at elevated levels and provide protection against subsequent MCMV infection (6–11).

The NK cell response to MCMV infection is the prototype system within which elements required to generate memory NK cells were first identified (12–17). Infection with human (H)CMV drives selective expansion of NK cells expressing the C-type lectin-like activating receptor NKG2C, prompting speculation that NKG2C on human NK cells functions in an analogous way to Ly49H on murine NK cells (18–21). In several HCMV-infected cell culture systems, selective expansion of NKG2C-expressing NK cells depends upon the presence of certain cytokines and interaction between NKG2C and HLA-E complexed with particular peptides (22, 23). Peptides derived from the HCMV UL40 protein enable selective proliferation of NKG2C-expressing NK cells, including those from HCMV naïve individuals (24). The NKG2C-expressing NK cells expanded *in vivo* or *in vitro* by exposure to HCMV acquire phenotypic changes that reflect an increased capacity for effector functions (25–27). This differentiation produces CD57^pos^ NK cells with increased CD16 expression, lower levels of the associated FcεRIγ (FcRγ) adaptor protein, reduced natural cytotoxicity receptor (NCR) expression, and epigenetic changes increasing the accessibility of cytokine promoter regions (25, 26, 28, 29). The CD57/NKG2C-expressing NK cells are reportedly more responsive to stimulation through CD16, at least in terms of antibody-dependent cytokine production (25–27).

Aging, and various forms of immunological stress, including congenital, iatrogenic, and HIV infection, exacerbate HCMV-driven expansion of NKG2C-expressing NK cells (21, 30–34). It is common for HIV/HCMV co-infected individuals to have large NK cell fractions expressing CD57 and NKG2C, within which limitations to NK cell adaptation imposed by terminal differentiation or exhaustion might be evident (34). Therefore, to assess NK cell function along a phenotypic spectrum of adaptation to HCMV infection, we studied healthy controls and HIV-infected individuals displaying varying degrees of NK cell adaptation. This included HCMV-infected and seronegative controls, an HIV-infected HCMV-seronegative group, an HIV/HCMV co-infected group with small fractions of NKG2C^pos^ NK cells and an HIV/HCMV co-infected group with large fractions of NKG2C^pos^ NK cells. Functional assessment began with exposure of NK cells from HCMV-seronegative controls to HCMV-related cytokines and extended across a wide range of NK cell exposure and adaption to HCMV infection, as indicated by the accumulated fractions of phenotypically adapted NK cells.

## Materials and methods

### Study subjects and sample collection

This study was carried out in accordance with the recommendations of the Canadian Tri-Council Policy Statement: Ethical Conduct for Research Involving Humans. The protocol was approved by the Health Research Ethics Authority of Newfoundland and Labrador, Canada. All subjects gave written informed consent in accordance with the Declaration of Helsinki. Whole blood was collected with informed consent from healthy donors and peripheral blood mononuclear cells (PBMC) isolated by Ficoll-Paque (VWR, Mississauga, ON, Canada) density gradient centrifugation were suspended in lymphocyte medium consisting of RPMI-1640 supplemented with 10% fetal calf serum (FCS), 200 IU/mL penicillin/streptomycin (P/S), 1% 1 M HEPES, 1% L-glutamine (all from Invitrogen, Carlsbad, CA, USA) and 2.0 × 10^−5^ M 2-mercaptoethanol (Sigma-Aldrich, St. Louis, MO, USA). Individuals infected with HIV recruited through the Newfoundland and Labrador Provincial HIV Clinic provided informed consent for whole blood collection, immunological studies, and researcher access to medical laboratory records. Freshly isolated PBMC were resuspended in freezing medium composed of lymphocyte medium supplemented to 20% FCS with 10% dimethyl sulfoxide and cooled at 1°C/min overnight to −80°C. Frozen PBMC were then maintained in liquid nitrogen until analysis. Cryopreserved PBMC were recovered overnight in lymphocyte medium at 37°C, 5% CO_2_. Humoral and CD8^pos^ T cell responses against CMV were measured previously as described and data included with general characteristics of the HIV-infected study subjects (34, 35).

### Generation of HCMVsn

MRC-5 cells (from Dr. Jules Doré, Memorial University of Newfoundland, St. John's, NL, Canada) were seeded at 1.25 × 10^5^ cells per well in a 24-well plate and grown in DMEM supplemented with 10% FCS, 1% L-glutamine, 1% P/S, and 1 mM sodium pyruvate (Invitrogen) at 37°C with 5% CO_2_. Forty-eight hours after seeding, MRC-5 cells were infected with either HCMV AD169 from Dr. Karen Biron through the NIH AIDS Reagent Program (NIAID, NIH) at multiplicity of infection (MOI) 0.025 for 1 h or a recombinant vaccinia virus expressing β-galactosidase (vSC8) at MOI 0.2, from Dr. Bernie Moss through the NIH AIDS Reagent Program or left untreated (36). Conditioned medium from uninfected (CONsn) and AD169-infected (HCMVsn) MRC-5 cells was harvested and medium replaced in 24 h increments post infection for a total of 120 h. Conditioned medium from vSC8-infected MRC-5 cells (vSC8sn) was harvested and medium replaced in 24 h increments post infection for a total of 72 h. Supernatants were clarified for 10 min at 400 *g* to remove cell debris, frozen at −80°C in single-use aliquots and recovered on ice. Inactivated HCMVsn was generated by collecting conditioned medium from MRC-5 cells that were exposed to the same amount of ultraviolet (UV)-irradiated AD169 (41 watts for 1 h at 30 cm).

### Cytotoxicity and redirected lysis assays

K562 (ATCC® CCL 243™), P815 (ATCC® TIB-64™), and HLA-B27-transfected C1R (C1R-B27; Dr. Kelly McDonald, University of Manitoba, Winnipeg, MB, Canada) cell lines were propagated in lymphocyte medium at 37°C with 5% CO_2_ and maintained in log phase for ^51^Cr labeling and cytotoxicity assays. K562, C1R-B27, or P815 cells were labeled for 90 min with 100 μCi ${\text{Na}}_{2}^{51}$CrO_4_ (PerkinElmer, Akron, OH, USA). C1R-B27 cells were incubated 30 min with 1 μg/mL monoclonal antibody (mAb) against pan HLA-I (W6/32, ATCC® HB-95™) to sensitize cells to antibody-dependent cellular cytotoxicity (ADCC). For interferon (IFN)-α/β receptor blocking experiments, PBMC were pretreated 30 min with 5 μg/mL of mAb (clone MMHAR-**2**) against the IFN-α/β receptor chain 2 (IFNAR) prior to their addition in a cytotoxicity assay as described (37). When indicated, purified active recombinant human IFN-α2 (US Biological, Salem, MA, USA) was used at a final concentration of 20 pg/mL. Receptor-triggered cytotoxicity was measured by adding 3 μg/mL soluble IgG isotype control (Ag8) or mAb (clones in parentheses) against human NKp30 (210845), NKG2C (134591), or NKG2D (149810) from R&D Systems (Minneapolis, MN, USA) and NKp44 (9E2) or NKp46 (P44-8) from BioLegend (San Diego, CA, USA). PBMC were incubated in microtiter plates in a final volume of 300 μL for 5 h with 5 × 10^3^ ^51^Cr-labeled K562, C1R-B27, or P815 target cells/well at various effector to target (E:T) ratios with either CONsn, vSC8sn or HCMVsn at a final dilution of 1 in 5. ^51^Cr release was measured in 125 μL supernatants collected from each well on a Wallac 1480 Wizard gamma counter. Control wells for spontaneous lysis contained target cells in medium alone while target cells in maximum lysis wells were treated with 1 N HCl. Percent specific lysis was calculated by (experimental ^51^Cr release – spontaneous ^51^Cr release)/(maximum ^51^Cr release – spontaneous ^51^Cr release) × 100.

### Macromolecular clearance of conditioned media

Intact HCMV particles were cleared from conditioned media by treating CONsn or HCMVsn with 0.06 μg/mL monoclonal anti-gB (Dr. Lucy Rasmussen, NIH AIDS Reagent Program) for 2 h at 4°C in a tube rotator followed by 30 min incubation with 4 × 10^6^ sheep anti-mouse IgG Dynabeads® (ThermoFisher) per milliliter of conditioned media. Beads and virus particles were pelleted for 1 min at 1,000 *g* after which time supernatants were decanted and stored at −70°C for functional experiments. To remove insoluble materials from conditioned media, samples were ultracentrifuged using a Sorvall TH-641 rotor at 100,000 *g* for 16 h at 4°C.

### Multiplex array

Concentrations of cytokines and chemokines within culture medium collected from MRC-5 cells untreated or infected with AD169 or vSC8 (see above) were measured by Milliplex (Millipore, Merck KGaA, Darmstadt, Germany) according to the manufacturer instructions and analyzed on the BioPlex-200 (Bio-Rad, Mississauga, ON, Canada). Samples were run in duplicate and incubated overnight to improve the sensitivity of detection as previously described (38).

### Flow cytometry

Human PBMC were stained using directly conjugated mAb against human CD3 (BW264/56), CD56 (REA196), CD57 (TB03), from Miltenyi Biotec (San Diego, CA, USA), Tim-3 (F38-2E2) from BioLegend and NKG2C (134591) from R&D Systems. Cells were stimulated with 1 μg anti-CD16 mAb (3G8; BioLegend) per 10^6^ PBMC and prepared for intracellular staining by adding brefeldin A (Sigma-Aldrich) 1 h after the start of incubation to a final concentration of 10 μg/mL and continuing the incubation for an additional 4 h. NK cell degranulation was detected by introducing directly conjugated anti-CD107a mAb (H4A3; BioLegend) at a 0.25 μg per 10^6^ PBMC at the time of brefeldin A addition. Cells were fixed and permeabilized after 5 h incubation using the Inside Stain Kit (Miltenyi Biotec) as per manufacturer's instructions and then stained with directly conjugated polyclonal Ab against human FcRγ from MilliporeSigma (Burlington, MA, USA) and anti-human IFN-γ mAb (4S.B3) from eBioscience (San Diego, CA, USA). Non-viable cells were excluded by fixable live/dead stain (Invitrogen) as per manufacturer's instructions. Data were acquired using a MoFlo Astrios EQ flow cytometer and data analyses and illustration performed using Kaluza software (both Beckman Coulter, Brea, CA, USA).

### Statistical analysis

Statistical analyses were performed using GraphPad Prism software version 5 with two-sided *p* < 0.05 considered significant. Normality of data distributions were assessed with Kolmogorov–Smirnov, Shapiro–Wilk and D'Agostino and Pearson tests. Student's paired or unpaired *t*-tests were used for group comparisons when data were normally distributed and non-parametric Wilcoxon paired signed-rank test or Mann–Whitney *U*-tests used otherwise as appropriate.

## Results

### Soluble factors released early in HCMV infection augment NK cell cytotoxicity

**Figure 1:**
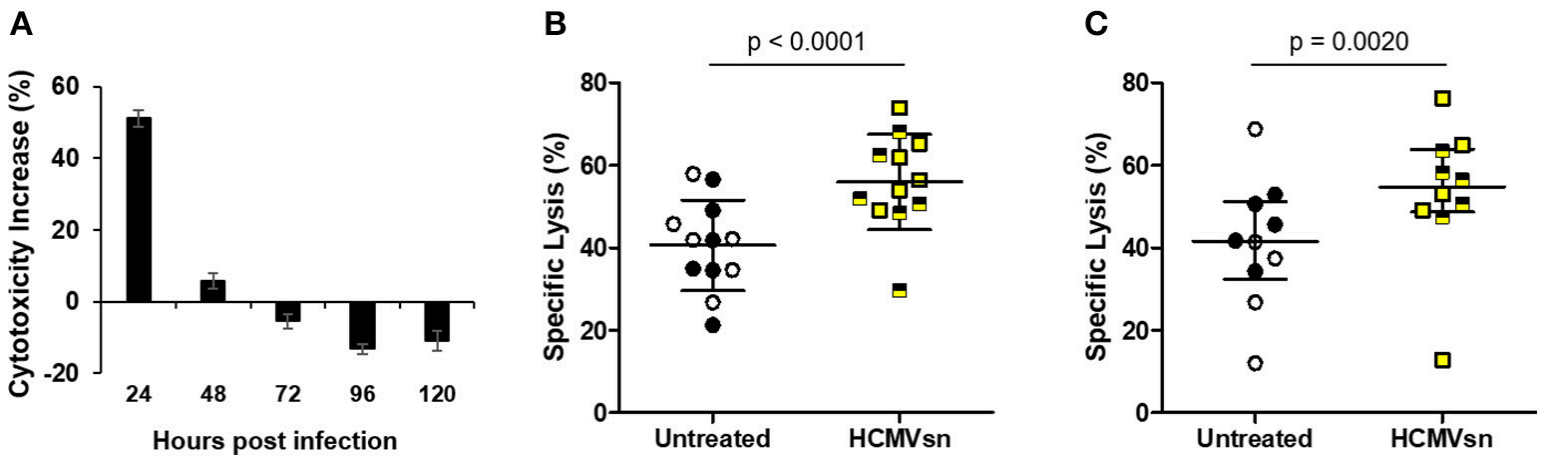
Effect of HCMVsn on NK cell cytotoxic responses of healthy controls. **(A)** CONsn or HCMVsn was collected at 24 h intervals up to 120 h post infection, diluted 1:5 and effects on NK cytotoxicity tested using fresh PBMC from healthy controls in 5 h ^51^Cr release assays. NK cell cytotoxicity with either CONsn or HCMVsn against **(B)** K562 cells (*n* = 12) or **(C)** anti-HLA-I-coated C1R-B27 cells (*n* = 10) was measured over 5 h at E:T 60:1. Subjects seropositive for HCMV are represented with filled black circles or yellow squares half-shaded black. Error bars in **(A)** represent mean ± standard deviation (SD) of two replicates, **(B)** mean ± SD with Student's paired *t*-test used for comparisons between conditions and **(C)** median with interquartile range (IQR) with Wilcoxon signed-rank test used to estimate the probability of a difference between conditions. Percent cytotoxicity increase in **(A)** was calculated as [(% specific lysis with HCMVsn – % specific lysis with CONsn)/% specific lysis with CONsn] × 100 from paired data performed in duplicate.

Natural killer cells are considered important effectors during herpesvirus infections. To examine how NK cells respond to factors released early in HCMV infection, we collected supernatant from uninfected (CONsn) and AD169-infected (HCMVsn) MRC-5 fibroblasts over 24 h intervals up to 120 h post infection. Supernatant was collected from vSC8-infected (vSC8sn) MRC-5 fibroblasts over 24 h intervals up to 72 h post infection. We then exposed NK cells to these supernatants in the presence of K562 target cells in 5 h ^51^Cr release assays to assess the effects of acute exposure on NK cell cytotoxicity. The vSC8sn collected from any of the three time points had no effect on NK cell cytotoxicity against K562 cells (data not shown, *n* = 3) compared to CONsn. In contrast, HCMVsn collected within the first 24 h after infection increased NK cell target killing by 50% relative to cytotoxicity in the presence of CONsn (Figure 1A). While this property of HCMVsn persisted up to 48 h post infection with conditioned media collected over a cumulative 48 h (data not shown), its production peaked during the first 24 h of infection as exposure to HCMVsn collected between 24 and 48 h post infection had little effect on NK cell activity (Figure 1A). Increased NK cell cytotoxicity following exposure to HCMVsn collected at 24 h occurred for all 12 donors tested, with a mean 38% boost in natural cytotoxicity (Figure 1B) and 29% increase in NK cell ADCC (Figure 1C). An equal number of HCMV-seropositive and seronegative donors, differentiated by symbol shading, are shown in Figure 1, with no apparent differences in responsiveness to stimulation with HCMVsn related to previous HCMV exposure. Factor(s) present in HCMVsn and primarily produced the first 24 h after infection cause a rapid significant increase in NK cell natural cytotoxicity and ADCC.

### HCMVsn augments cytotoxicity triggered through multiple NK cell activating receptors

**Figure 2:**
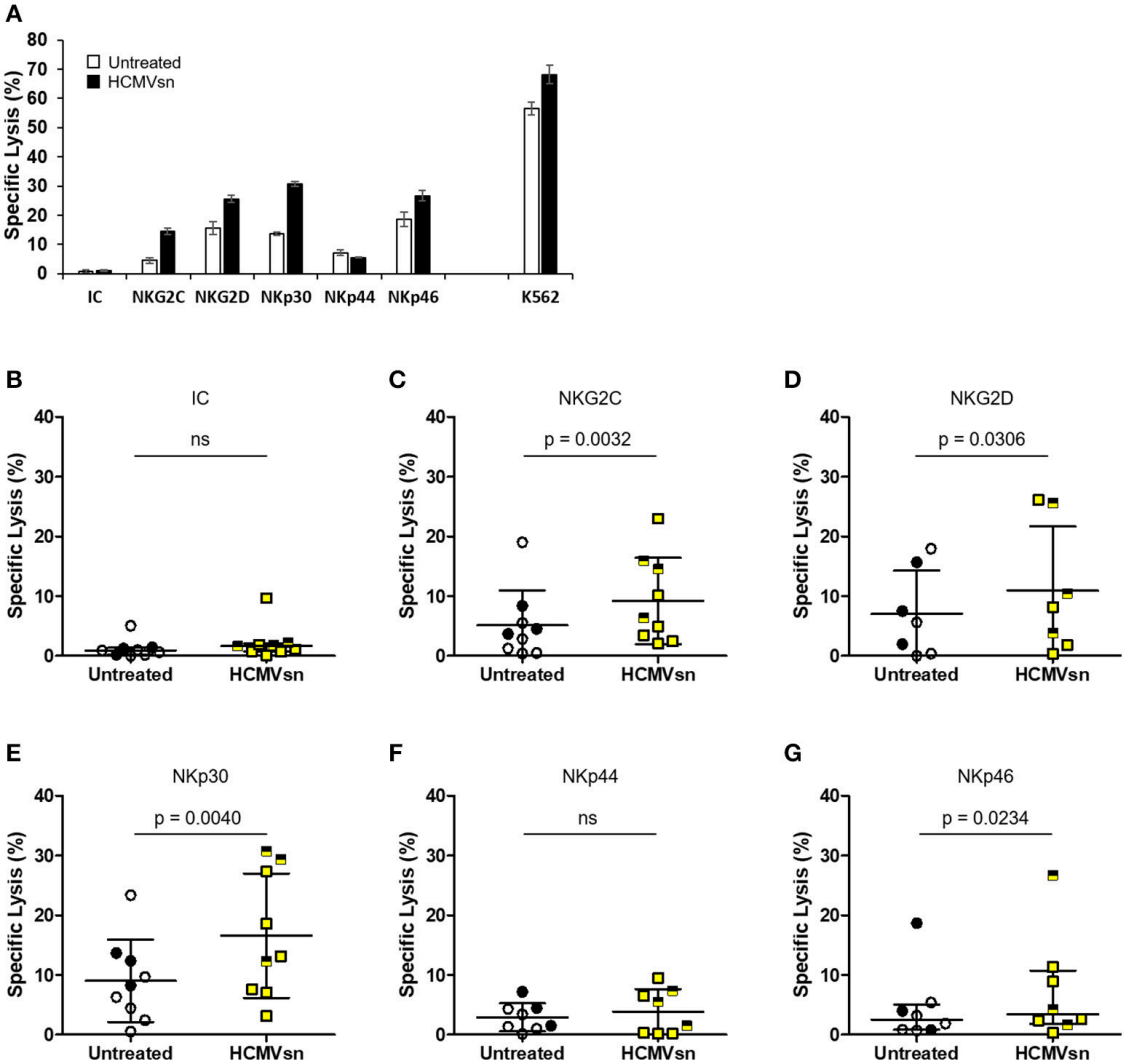
Effect of HCMVsn on NK cell cytotoxicity directed through different receptors. **(A)** Natural cytotoxicity (K562 cells), and redirected lysis of P815 cells in the presence of NK receptor-specific mAb for a representative subject with or without HCMVsn was measured over 5 h. Error bars represent mean ± standard error of the mean with three replicates. Redirected P815 cell lysis was measured at E:T 30:1 with 3 μg/mL **(B)** isotype control (IC), **(C)** anti-NKG2C, **(D)** anti-NKG2D, **(E)** anti-NKp30, **(F)** anti-NKp44, or **(G)** anti-NKp46 mAbs. Assays represented in plots **(B–G)** were performed in triplicate for 7–9 individuals. Subjects seropositive for HCMV are represented with filled black circles or yellow squares half-shaded black. Error bars for isotype control and anti-NKp46 plots represent median with IQR and the probability of a difference between conditions was assessed by Wilcoxon signed-rank test. All other bars represent mean ± SD with comparison between conditions by Student's paired *t*-test.

As HCMVsn increased both NK cell natural cytotoxicity and CD16-dependent responses, we investigated the effect of HCMVsn on cytotoxicity through other common NK cell activating receptors. We probed NKG2C, NKG2D, NKp30, NKp44, and NKp46 using a redirected lysis assay in which murine FcR-expressing P815 cells orient mAb specific for particular activating receptors to enable selective NK cell activation. A representative summary of target cell lysis by natural cytotoxicity, ADCC and redirected killing through individual activating receptors is shown in Figure 2A. Using an IgG isotype control antibody, there was little P815 cell lysis and no significant difference from control conditions when HCMVsn was present during the 5 h assay (Figure 2B). By introducing mAb against individual NK cell activating receptors, we observed that HCMVsn significantly increased P815 cell lysis through NKG2C (Figure 2C), NKG2D (Figure 2D), and the NCRs NKp30 and NKp46, but not NKp44 (Figures 2E–G). These data demonstrate that HCMVsn sensitizes NK cells for cytotoxicity triggered through CD16, most NCRs and through activating C-type lectin-like receptors. As with the increased killing of K562 and ADCC, there was no apparent difference in the responses of NK cells from HCMV-seropositive and seronegative subjects discriminated on the graphs by different symbols.

### IFN-α produced during HCMV infection enhances NK cytotoxicity

**Figure 3:**
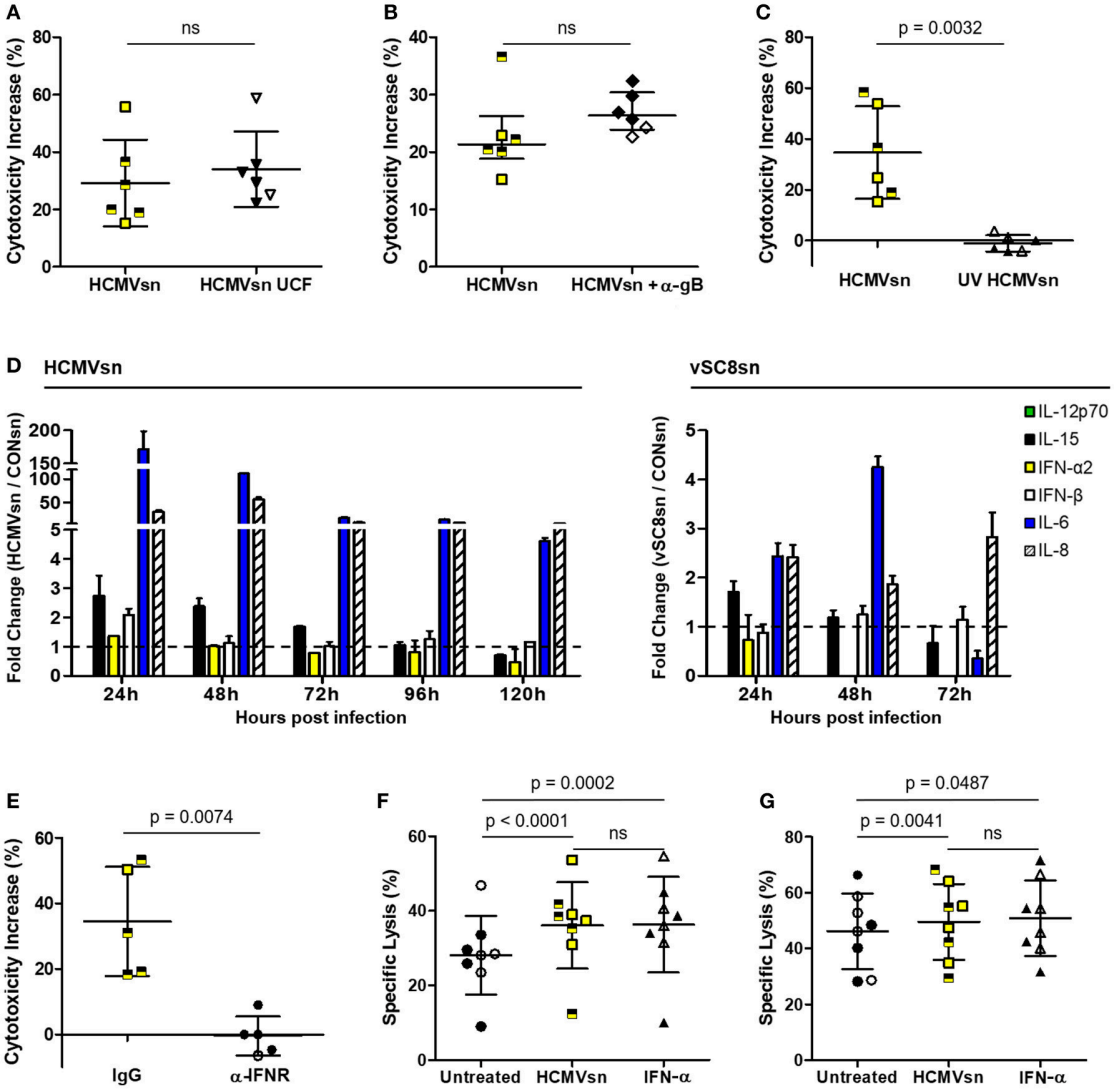
Identification of cytokines produced by HCMV-infected MRC-5 cells. The effect of HCMVsn on NK cell cytotoxicity was assessed by ^51^Cr release using K562 target cells at E:T 30:1 after removal **(A)** of insoluble materials by ultracentrifugation (HCMVsn UCF) or **(B)** HCMV particles with monoclonal anti-gB and goat anti-mouse IgG Dynabeads. **(C)** Ultraviolet-light inactivated virus was used to generate UV HCMVsn and its effect on NK cell cytotoxicity was measured as above (*n* = 6). Subjects seropositive for HCMV are represented with filled black circles or triangles and yellow squares half-shaded black. Error bars in **(A,C)** represent mean ± SD with conditions compared using Student's paired *t*-test and **(B)** median with IQR and conditions compared by Wilcoxon signed-rank test. **(D)** Luminex analysis of HCMVsn collected at 24 h intervals over 120 h (left panel) or vSC8sn collected at 24 h intervals over 72 h (right panel) compared IL-6, IL-8, IL-15, IL-12p70, IFN-α2, and IFN-β concentrations. Data shown represents fold change in the amount of cytokine detected in HCMVsn or vSC8sn/CONsn performed in duplicate ± SD. **(E)** PBMC (*n* = 5) were pretreated with 5 μg/ml of IgG control or anti-IFNAR before incubation with CONsn or HCMVsn in a 5 h ^51^Cr release assay. Bars represent mean ± SD performed in duplicate with conditions compared using Student's paired *t*-test. The effect of 20 pg/mL recombinant IFN-α2 was compared to that of HCMVsn on NK cell **(F)** natural cytotoxicity and **(G)** ADCC (*n* = 8). Bars represent mean ± SD, performed in triplicate with conditions compared using Student's paired *t*-test. Data in **(A–C,E–G)** represent percent cytotoxicity increase or percent specific lysis, respectively, calculated as described in the legend for Figure 1.

While a cell-free component of HCMVsn increased NK cell pan cytotoxic function, it was unclear whether this effect was mediated through macromolecular interactions with different NK cell activating receptors or by cytokines. Augmented NK cell cytotoxicity was sustained following clearance of macromolecular material by HCMVsn ultracentrifugation (Figure 3A) or following selective removal of HCMV particles by magnetic beads and a mAb against HCMV glycoprotein gB (Figure 3B). Inactivating HCMV before its addition to MRC-5 cells (UV HCMVsn) prevented the increase in NK cell cytotoxicity observed with replication competent HCMVsn (Figure 3C), indicating that a soluble product from HCMV-infected cells increases NK cell cytotoxicity through multiple activating receptors.

To identify potential factors present in HCMVsn that increase NK cell cytotoxicity, we measured analytes using a bead-based multiplex assay. Luminex analysis of HCMVsn revealed increased concentrations of monocyte chemoattractant protein and macrophage colony stimulating factor (data not shown), IL-6, IL-8, IL-15, IFN-α2, and IFN-β compared to CONsn (Figure 3D right and left panels). Modest concentrations of biologically active IL-15 (10 pg/mL; data not shown) were also detected. Analysis of vSC8sn revealed increased concentrations of IL-6, IL-8, and IL-15 with a marginal increase in IFN-β at the 48 h time point. Blocking stimulation through IFNAR alone with a specific mAb fully prevented any increase in NK cell activity in response to HCMVsn (Figure 3E). This blocking effect was specific for type I IFNs as increased NK cell activity in response to IL-2 was maintained in the presence of the same amount of mAb against IFNAR (data not shown). HCMV-infected fibroblasts produced both IFN-α2 and IFN-β (80 and 450 pg/mL, respectively, data not shown) during the first 24 h of infection. To determine whether the amount of IFN-α2 detected in HCMVsn during the first 24 h of infection could alone mediate the same increases in NK cell cytotoxicity as HCMVsn, we compared NK cell natural and antibody-dependent cytotoxic activity in response to HCMVsn or purified recombinant IFN-α2. Exposure to similar concentrations of IFN-α2 (20 pg/mL) as added with HCMVsn diluted 1 in 5 in ^51^Cr release assays increased NK cell natural cytotoxicity (Figure 3F) and ADCC (Figure 3G) to an extent indistinguishable from the increases mediated by HCMVsn. Thus, stimulation through the type I IFN receptor alone rapidly sensitized NK cells to natural and antibody-dependent cytotoxicity and the acute increase in natural cytotoxicity and ADCC for NK cells from healthy controls mediated by soluble factors in HCMVsn is replicated with IFN-α2 alone at levels present in HCMVsn.

The increase in NK cell cytotoxicity following exposure to HCMVsn occurred with both HCMV^pos^ and HCMV^neg^ healthy donors indicating that the NK cell response to cytokines produced by HCMV-infected cells is independent of previous sensitization from *in vivo* HCMV infection (Figures 1, 2). To investigate such responses along a continuum of NK cell adaptation to HCMV infection, we tested effects of HCMVsn and IFN-α2 with HIV-infected subjects whose NK cells spanned a broad range of adaptation in response to HCMV infection.

### HCMVsn and IFN-α2 enhance NK cell ADCC in HIV-infected individuals

After establishing that NK cells from healthy donors respond rapidly, robustly and independent of HCMV serostatus to IFN-α2 produced by HCMV-infected fibroblasts, we next studied NK cell responses from HCMV^neg^ and HCMV^pos^ individuals within an HIV-infected study cohort. Since adapted NK cells exhibit enhanced CD16-mediated effector responses, we measured the impact of HCMVsn and IFN-α2 on NK cell ADCC in HIV infection and compared baseline levels of ADCC between three groups reflecting a broad range of NK cell adaptation to HCMV infection. General characteristics of the individuals and comparisons between HCMV-seronegative and seropositive groups are shown in Table 1. We selected three groups of HIV-infected study subjects, two distinguished by high (>20%) vs. low (<6%) fractions of NK cells expressing NKG2C and a third distinguished by seronegative HCMV status (Table 1). Nine of the 28 age- and sex-matched HIV-infected individuals selected for this study were HCMV-seronegative. The efficacy of antiretroviral therapy was roughly equivalent between groups as indicated by undetectable HIV viral loads (<50 copies HIV RNA/mL plasma). Neither nadir, nor current CD4^pos^ T cell counts, indicative of past disease progression and present immunological status, respectively, differed significantly between groups. All donors had a robust fraction of mature (CD57^pos^) NK cells, independent of HCMV status or fraction of their NK cells expressing NKG2C. However, the percentage of NK cells expressing CD57 was significantly lower in the NKG2C^lo^ group than in the NKG2C^hi^ group (*p* = 0.0133, Mann–Whitney *U*-test), suggesting lesser overall NK cell maturation in the NKG2C^lo^ group (Table 1). There were no significant differences in either humoral (anti-CMV IgG levels) or cellular (% CD8^pos^ T cells specific for CMV pp65 and IE-1) immune responses against CMV between the HCMV^pos^ groups with high or low fractions of NK cells expressing NKG2C.

**Table 1:** HIV-infected study subject characteristics.

	**Age range**	**Sex**	α-CMV IgG (OD)^*^	**CMV-specific CD8^pos^ T cells (%)^†^**	**CD4^pos^ T cells^‡^**	**Nadir^§^**	**HIV VL^||^**	**NKG2C^pos^ NK cells (%)**	**FcRγ^neg^ NK cells (%)**	**CD57^pos^ NK cells (%)**	**Tim-3^pos^ NK cells (%)**
**HCMV**^neg^	44–56	7♂ 2♀	0.054	0.1	714	51	1.4	1.03	3.95	22.54	2.36
			0.055	0.1	640	286	2.3	2.68	22.19	11.89	8.22
			0.036	0.0	1320	81	1.3	2.84	7.07	54.79	5.31
			0.044	0.0	700	324	1.3	2.35	12.32	20.77	8.28
			0.029	0.1	966	154	1.3	2.99	7.63	57.54	0.53
			0.048	0.0	429	85	1.3	3.70	9.43	55.95	5.55
			0.080	0.0	667	154	1.3	4.41	20.72	54.68	4.95
			0.027	0.0	912	658	1.3	3.31	7.35	50.74	6.56
			0	0.0	833	490	1.3	2.37	30.07	61.86	0.40
**x̄**	51.8		0.040	0.0	798	254	1.4	2.85	13.41	43.42	4.68
**HCMV**^pos^ **NKG2C**^lo^	33–58	7♂ 2♀	1.265	4.7	1085	314	1.3	1.96	61.14	41.84	3.58
			1.418	2.2	638	192	1.7	4.76	55.75	26.74	4.2
			1.324	5.0	946	245	1.3	1.46	12.60	54.96	6.61
			0.686	1.0	1026	229	1.3	0.30	6.74	37.88	3.8
			1.047	2.2	492	93	1.3	4.72	12.76	26.46	6.69
			1.567	1.6	624	6	1.3	0.47	25.97	32.04	5.64
			0.846	2.2	957	192	1.6	5.92	26.07	39.83	8.56
			0.767	0.7	725	660	1.3	1.72	19.23	58.60	8.37
			0.670	1.9	903	169	1.3	1.81	71.70	10.50	6.25
**x̄**	47.8		1.070	2.4	822	233	1.4	2.57	32.44	36.54	5.97
**HCMV**^pos^ **NKG2C**^hi^	36–60	8♂ 2♀	1.552	1.6	588	25	1.3	46.25	46.25	42.97	6.82
			0.897	0.2	570	206	1.6	44.21	86.07	79.36	4.25
			1.489	1.0	285	16	ND	50.81	80.85	45.91	7.03
			0.993	0.8	760	380	1.3	41.07	47.98	68.19	8.34
			0.810	0.6	780	245	1.3	27.05	77.83	54.35	3.24
			0.723	0.4	760	63	1.3	24.4	30.63	40.37	9.68
			1.769	0.2	1254	276	1.3	27.61	44.42	39.01	7.02
			0.579	7.2	792	108	1.3	46.48	60.22	61.39	2.74
			1.275	2.3	748	407	1.3	42.66	42.07	66.01	6.05
			1.676	4.0	455	416	1.3	42.85	57.09	52.93	5.53
**x̄**	48.1		1.180	1.9	699	214	1.33	39.34	57.34	55.05	6.07

* *Optical density (OD) by ELISA measuring α-CMV IgG (plasma diluted 1:500) against lysate from HCMV AD169-infected MRC-5 cells (34)*.

† *Percentages of CMV-specific CD8^pos^ T cells identified by stimulation with overlapping peptides from CMV pp65 and IE-1 proteins followed by detection of intracellular IFN-γ (34, 35)*.

‡ *Number of CD4^pos^ T cells per microliter of peripheral blood at time of testing*.

§ *Lowest recorded CD4^pos^ T cell count per microliter of peripheral blood*.

|| *Log_10_ copies HIV RNA per milliliter of plasma at time of testing*.

*ND, not determined*.

**Figure 4:**
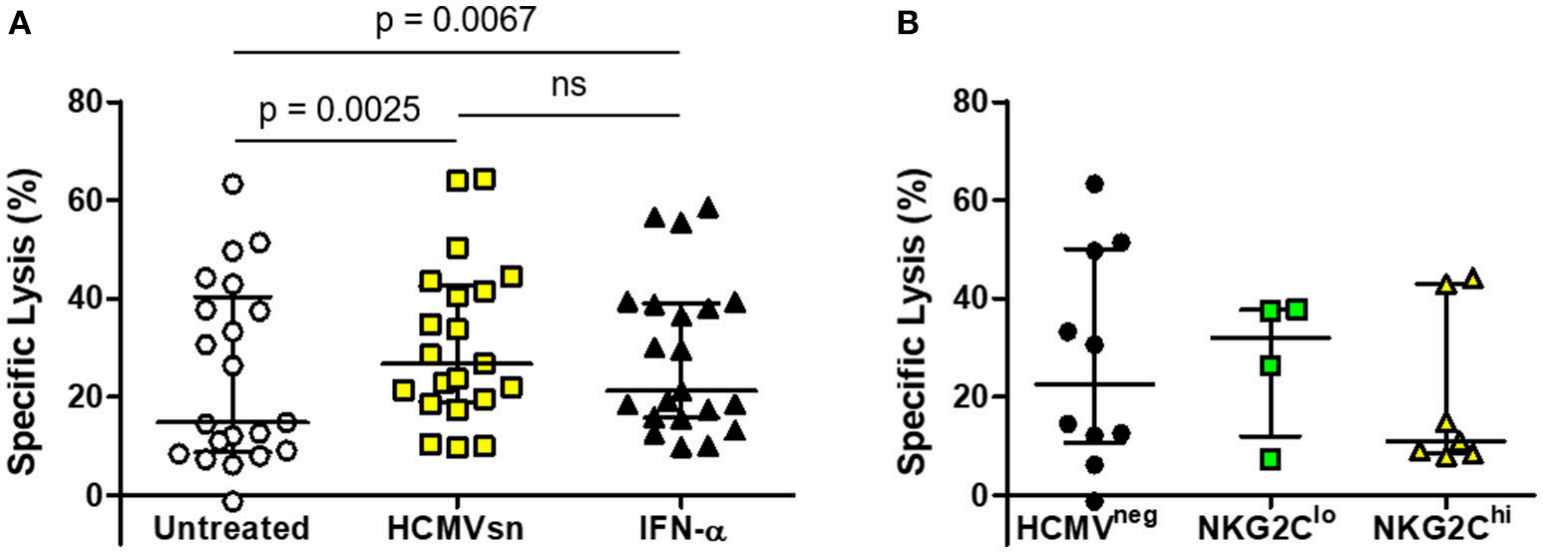
Effects of HCMVsn and IFN-α2 on ADCC of NK cells from HIV-infected individuals. **(A)** ADCC within the HIV-infected group was measured in ^51^Cr release assays over 5 h at E:T 30:1 in the presence of a 1:5 dilution of HCMVsn or 20 pg/mL recombinant human IFN-α2 as previously described and compared to untreated conditions (*n* = 21). **(B)** Baseline ADCC levels were compared between the three groups of HIV-infected individuals distinguished by HCMV serostatus and fraction of NK cells expressing NKG2C. Error bars represent median with IQR, performed in duplicate with conditions compared using Wilcoxon signed-rank test.

The HCMV^neg^ individuals and those with high or low fractions of NKG2C-expressing NK cells were pooled to assess responsiveness of NK cells from HCMV^neg^ and HCMV^pos^ subjects infected with HIV to HCMV-related cytokines. The mean increase in ADCC mediated by HCMV-related cytokines (24%) was similar to that seen in the HIV-naïve group and was not significantly different from the mean 14% increase elicited by recombinant IFN-α2 alone (Figure 4A). Neither HIV-infection nor adaptation to HCMV infection appeared to affect NK cell responsiveness with acute exposure to IFN-α2. Median levels of NK cell ADCC measured from HIV-infected individuals were also unrelated to either HCMV serostatus or NK cell adaptation to HCMV infection (Figure 4B). Adaptation to HCMV infection had little effect on the capacity of NK cells from HIV-infected individuals to respond to acute IFN-α2 exposure or to mediate ADCC.

### Long term adaptation of NK cells from HIV-infected individuals to HCMV infection

**Figure 5:**
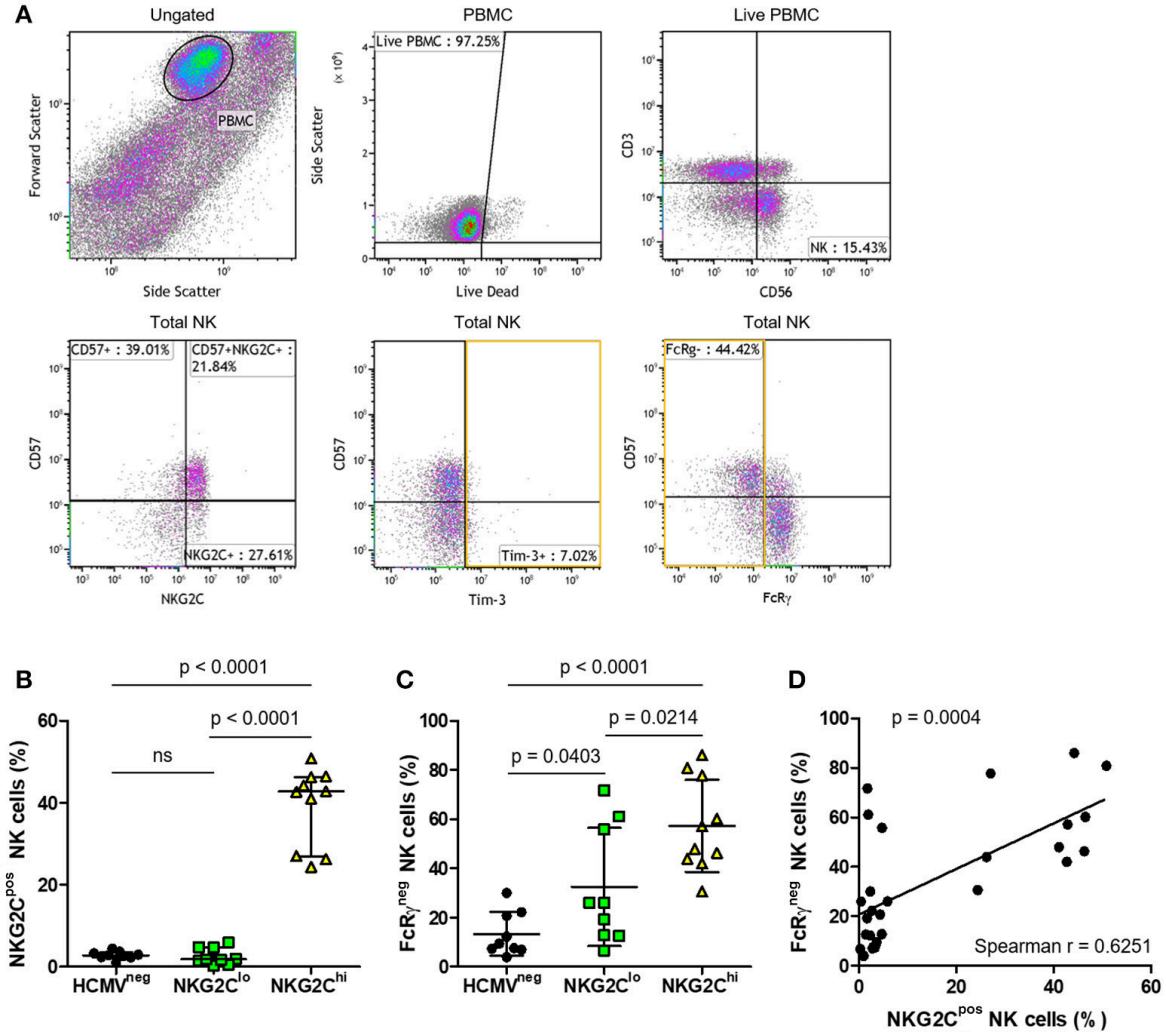
Phenotypic characterization of NK cells from HIV-infected individuals with different levels of adaptation to HCMV infection. To identify the NK cell population, **(A)** PBMC were selected and dead cells excluded. NK cells were identified by expression of CD56 in the absence of CD3 and further characterized for percentage expressing NKG2C, CD57, Tim-3, and FcRγ in a representative plot. Summary plots depict the percentage of total NK cells that are positive for **(B)** NKG2C or **(C)** FcRγ. Individual donors were grouped based on HCMV serostatus (HCMV^neg^) and fraction of NK cells expressing NKG2C within the HCMV^pos^ donors (NKG2C^lo^ or NKG2C^hi^). Correlation between NK cell NKG2C and FcRγ expression was assessed in **(D)** with Spearman correlation coefficient (r) calculated and the probability of a significant correlation (p) shown on the graph. Error bars in **(B)** for the HCMV^neg^ group represent median with IQR, while all others represent mean ± SD. Groups were compared using Mann–Whitney *U*-tests.

To study longer term effects of HCMV infection, including the possibility of progression to exhaustion, we compared features of the three groups of HIV-infected subjects distinguished by high vs. low NK cell fractions expressing NKG2C or by seronegative HCMV status (Table 1). The flow cytometry gating strategy for a representative HIV/HCMV co-infected donor is shown in Figure 5A. Median NKG2C expression levels were similar between the HCMV^neg^ and NKG2C^lo^ groups (Figure 5B), however, the influence of adaptation to HCMV within the NKG2C^lo^ group was evident from the lower mean fraction of NK cells expressing FcRγ (Figure 5C) and strong correlation between loss of FcRγ expression and level of HCMV-specific antibodies (Spearman *r* = 0.5539, *p* = 0.0022 Table 1). Loss of the FcRγ signaling adaptor subunit in parallel with expansion of NK cells expressing NKG2C is apparent from the progressive increase in percentage of FcRγ^neg^ NK cells across the three groups and by the significant correlation between fractions of NK cells expressing NKG2C and fractions lacking FcRγ (Figures 5C, D). Despite this general progression and significant correlation, three HIV-infected individuals within the NKG2C^lo^ group with <6% of their NK cells expressing NKG2C and >50% lacking FcRγ illustrate that loss of FcRγ by NK cells is not inexorably linked to NKG2C expression (Figure 5C, Table 1).

### Preservation of NK cell antibody-dependent effector functions in HIV-infected subjects

An association between NK cell adaptation to HCMV infection and increased capacity for antibody-dependent IFN-γ production was previously reported (39, 40). Since ADCC responses against antibody-coated C1R-B27 cells by the HIV-infected subjects measured by ^51^Cr release assays were similar, irrespective of HCMV status and NKG2C expression levels, we next measured and compared IFN-γ production and CD107a expression of NK cells in the three groups in response to CD16 stimulation.

**Figure 6:**
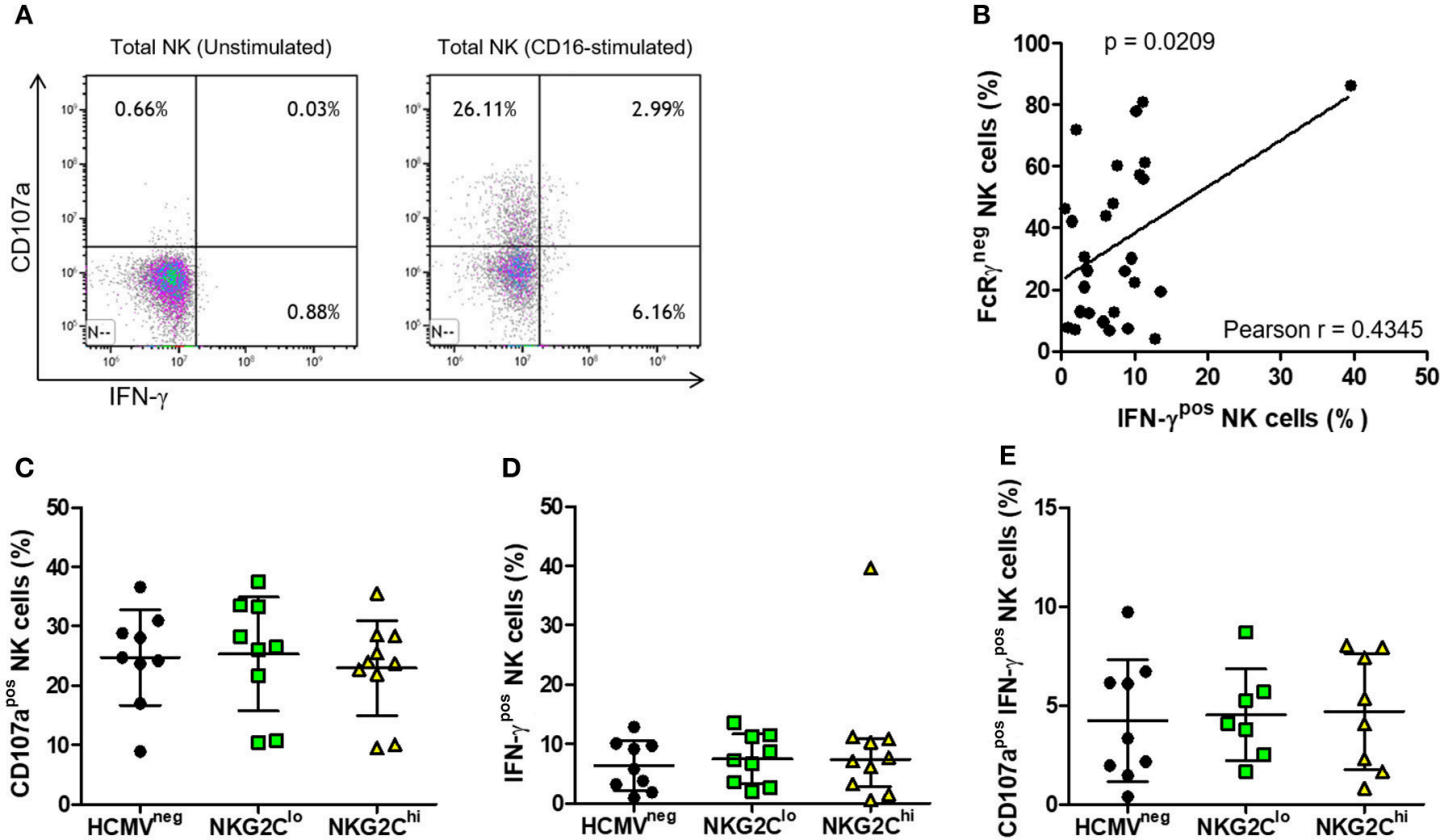
Effects of NK adaptation to HCMV infection on CD16-dependent degranulation and IFN-γ production in HIV infection. NK cells were untreated or stimulated for 5 h with anti-CD16 (3G8) after which time IFN-γ production and CD107a expression was assessed by flow cytometry. Representative plots with gating on total NK cells as in Figure 5 show **(A)** IFN-γ and CD107a expression on resting (left panel) and CD16-stimulated NK cells (right panel). For each donor, percent NK cells positive for **(B)** IFN-γ was plotted vs. percent FcRγ^neg^ cells and correlation between the magnitude of NK cell IFN-γ responses and fraction of NK cells lacking FcRγ was assessed using Pearson correlation coefficient (r) with the probability (p) of a significant correlation shown on the graph. The percentage of NK cells positive for **(C)** CD107a, **(D)** IFN-γ, or **(E)** doubly positive for CD107a and IFN-γ was measured for each donor (*n* = 7–10) and grouped as in Figure 5. Error bars represent mean ± SD where data was normally distributed and median with IQR in all other cases.

Activation through CD16 induced robust NK cell IFN-γ responses (representative plots in Figure 6A), the magnitude of which directly correlated with the percentage of NK cells lacking FcRγ (Figure 6B). This is exemplified by one subject with over 80% NK cells lacking FcRγ and over 40% producing IFN-γ in response to stimulation through CD16 (Figure 6B). In contrast, we found no significant correlation between the fraction of FcRγ^neg^ NK cells and fraction of NK cells expressing NKG2C or between NK cell FcRγ expression and degranulation, as measured by CD107a expression following CD16 stimulation. When we compared CD16-stimulated degranulation and IFN-γ production between HCMV^neg^, NKG2C^lo^, and NKG2C^hi^ groups, there were no significant differences in percentages of CD107a^pos^ NK cells (Figure 6C), IFN-γ^pos^ NK cells (Figure 6D) or polyfunctional NK cells doubly positive for CD107a and IFN-γ (Figure 6E). All groups' NK cells responded robustly to CD16 stimulation, irrespective of NKG2C expression levels or HCMV serostatus, illustrating HCMV-independent preservation of antibody-dependent NK cell responses in chronic HIV infection.

To assess NK cell progression toward an exhausted phenotype, expression of PD-1, LAG-3, and Tim-3 was assessed on NK cells within the three defined groups. Levels of PD-1 and LAG-3 were low to undetectable on NK cells (data not shown), but substantial fractions of NK cells within each group expressed Tim-3 (Figure 5A, Table 1).

### Antibody-dependent effector functions of Tim-3^pos^ NK cells from HIV-infected subjects

**Figure 7:**
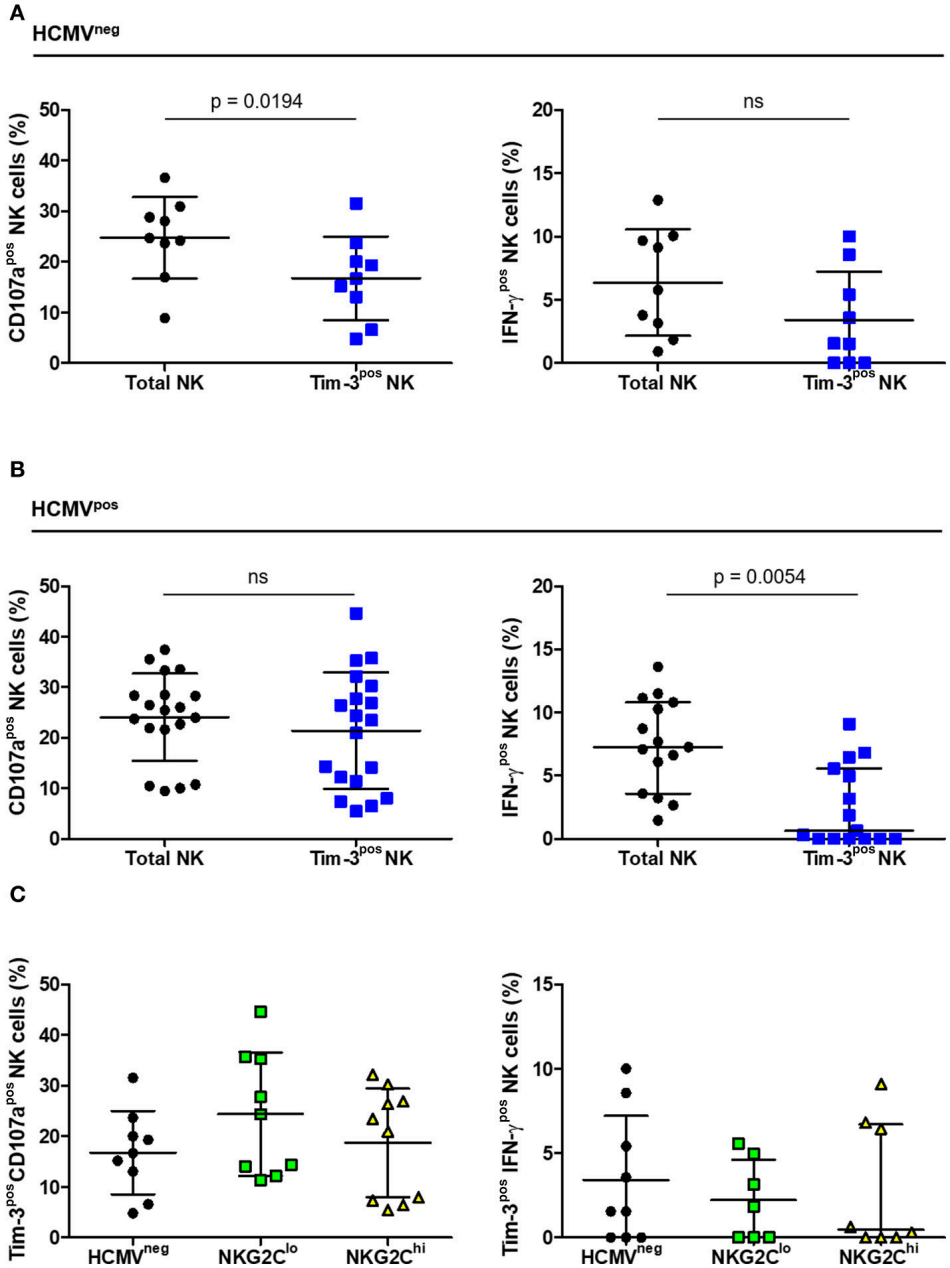
CD16-dependent degranulation and IFN-γ production by Tim-3^pos^ NK cells in HIV infection. The percentage of cells expressing CD107a (left panels) or IFN-γ (right panels) within either the total or Tim-3^pos^ NK cell populations after CD16 stimulation was measured based on the gating strategy shown in Figure 5 and contrasted between **(A)** HCMV^neg^ (*n* = 9) and **(B)** HCMV^pos^ (*n* = 15–19) and between the **(C)** HCMV^neg^, NKG2C^lo^, and NKG2C^hi^ HIV-infected study groups. Error bars in **(A)** represent mean ± SD and comparison between conditions carried out with Student's paired *t*-test. Bars in **(B)** represent mean ± SD and Student's paired *t*-test performed (left panel) or median with IQR with significance calculated by Wilcoxon signed-rank test (right panel). Error bars in **(C)** represent mean ± SD where data was normally distributed and median with IQR in all other cases.

As Tim-3 receptors were expressed at levels readily measurable by flow cytometry, we compared the extent of Tim-3 expression on NK cells within the three groups and the functional capacity of the Tim-3^pos^ subset. The percentage of NK cells expressing Tim-3 ranged from 0 to 10%, did not differ significantly between groups (Table 1) and CD16-stimulation did not induce NK cell Tim-3 expression (data not shown). The percentage of Tim-3^pos^ NK cells responding to CD16 stimulation with IFN-γ expression, CD107a expression, or both, was compared with the percentage of total NK cells responding likewise. Tim-3-expressing NK cells from HCMV^neg^ HIV-infected donors degranulated to a lesser extent than the general NK cell population but showed no apparent deficit in IFN-γ production (Figure 7A). In contrast, Tim-3^pos^ NK cells from HIV/HCMV co-infected donors responded similarly to the general NK cell population in terms of CD107a expression, but were less likely to produce IFN-γ in response to stimulation through CD16 (Figure 7B). The significance of Tim-3 expression on NK cells may vary with respect to the function studied and extent of adaptation to HCMV infection. There were no significant differences between HCMV^neg^, NKG2C^lo^, and NKG2C^hi^ groups in percentage of Tim-3^pos^ NK cells expressing CD107a or IFN-γ in response to stimulation through CD16 Figure 7C.

## Discussion

Expansion of NKG2C-expressing NK cells was initially reported in the context of multiple viral infections, including hepatitis C virus, HIV, chikungunya, and hantavirus, but subsequent investigation identified HCMV infection as the critical common denominator (41, 42). While only a small fraction of NK cells in healthy HCMV-seronegative individuals usually express NKG2C, HCMV infection triggers various levels of adaptation, resulting in more than 80% NKG2C^pos^ NK cells in extreme cases (34). Therefore, we envision a continuum of NK cell adaptation to HCMV infection through which naïve NK cells respond to initial HCMV exposure, differentiate into mature effector cells and, under the influence of time, repeated exposure and other factors, progress toward terminal differentiation. In this study, we investigated NK cell function along this proposed continuum, beginning with exposure of naïve NK cells to cytokines present during acute HCMV infection and encompassing responses of NK cell populations reflecting broadly different degrees of adaptation to HCMV. To compare the quality of NK cell responses across the spectrum of adaptation to HCMV, we selected subjects from a well characterized cohort of HIV-infected individuals within which there are HCMV-seronegative subjects, HCMV-seropositive subjects with small fractions of NKG2C^pos^ NK cells and HCMV-seropositive subjects with large fractions of NKG2C^pos^ NK cells (34). This chosen study group allowed comparison of CD16-dependent NK cell functions over a broad range of phenotypic adaptation to HCMV infection in the context of co-infection with HIV.

Acute exposure to conditioned media collected from HCMV-infected fibroblasts elevated NK cell cytotoxicity through NCRs, C-type lectin receptors and CD16, irrespective of NK cell donor HIV or HCMV status. While enhanced antibody-dependent effector function through CD16 is a characteristic of the NKG2C^pos^ NK cells adapted to HCMV infection, these adapted NK cells are also reported to downregulate NCRs (18). Downregulation of NCRs is a common consequence of chronic viral infection, likely involving physical interactions and chronic cytokine stimulation. In combination with additional stimulation, pan increases in NCR-mediated cytotoxicity stimulated by HCMV-related cytokines may lend toward phenotypic alterations over periods of prolonged cytokine exposure. While IL-6, IL-8, and IL-15 were present in HCMVsn together with type I IFNs, these same cytokines were also elevated in vSC8sn, which did not increase NK cell cytotoxicity, indicating that at most, they may play some ancillary role in acute activation of NK cell cytotoxicity. Our data indicate that IFN-α2 is sufficient to effect the same increases in NK cell cytotoxicity as mediated by conditioned media from HCMV-infected fibroblasts. There were no significant differences between NK cell responses to purified recombinant IFN-α2 vs. HCMVsn in either the healthy control cohort or HIV-infected group. As type I IFNs are produced in a variety of viral infections that do not lead to expansion of NKG2C^pos^ NK cells, it is unlikely that acute exposure to IFN-α2 alone contributes significantly to the NK cell adaptation specific to HCMV infection. Infection with HCMV *in vivo* may result in different patterns of type I IFN production and cellular responses dependent upon the local environment, cell type infected and virus characteristics.

By selecting three groups of individuals with comparable features of HIV infection, we were able to focus on the impact that different levels of adaptation to HCMV infection have on NK cell phenotype and function in this setting. Two HCMV-infected groups were selected for high vs. low levels of NKG2C expression, with the low NKG2C expression group indistinguishable from the HCMV^neg^ group in this respect. Although one donor within the NKG2C^lo^ category had a CD57^pos^ NK cell fraction comparable to those in the NKG2C^hi^ group, the rest had low to moderate (10–27%) CD57^pos^ NK cell fractions. The influence of HCMV infection on FcRγ downregulation was the most apparent phenotypic aspect, displaying a clear hierarchy; the HCMV^neg^ group had the lowest fraction of FcRγ^neg^ NK cells followed sequentially by the NKG2C^lo^ and NKG2C^hi^ groups. A disconnect between NKG2C expression and loss of FcRγ was notable in three individuals with <6% NKG2C^pos^ and more than 50% FcRγ^neg^ NK cells. Although it involves only three individuals in this case, the disconnect has also been noted in studies of NKG2C^null^ cohorts, where lack of NKG2C had little effect on HCMV-driven NK cell maturation (43–45). Despite these three outliers, there was a strong overall correlation between the fraction of NK cells expressing NKG2C and fraction of FcRγ^neg^ NK cells. Thus, NK cells from these outliers may express features similar to those from NKG2C^null^ subjects (45). Evidently, loss of FcRγ is a more consistent indicator of NK cell differentiation and adaptation in response to HCMV infection than NKG2C expression.

Adaptation to HCMV infection through NKG2C^pos^ NK cell expansion and loss of FcRγ reportedly produces an NK cell population with superior CD16-mediated effector functions (25–27). In the context of our HIV-infected study cohort, there was no evidence of this in cytotoxicity assays against antibody-coated target cells. Although we saw significant correlation between the size of the IFN-γ response and fraction of NK cells lacking FcRγ, there was also no significant difference between the groups in terms of the mean fraction of NK cells producing IFN-γ in response to CD16 signaling. Despite low levels of NKG2C^pos^FcRγ^neg^ NK cells, the HCMV^neg^ group had CD16-mediated responses equally as robust as the other groups and the HCMV^pos^NKG2C^lo^ group displayed no functional deficits relative to either of the other groups. This finding is somewhat unexpected in light of epigenetic remodeling of the NK cell *IFNG* locus following HCMV infection, with the adapted NK cell population reportedly exhibiting enhanced IFN-γ responses (39, 40). Whether this apparent discrepancy reflects the impact of HIV infection, different experimental methodology or peculiarities of the limited number of subjects tested in this study remains to be determined.

The extent of NK cell degranulation following CD16-stimulation was also not significantly different between groups and in contrast to IFN-γ production, we noted no significant correlation between the magnitude of CD107a responses and fraction of NK cells lacking FcRγ. Polyfunctional NK cell responses, indicated by dual expression of IFN-γ and CD107a also did not differ between groups. Thus, our data suggest that in the context of HIV infection, phenotypic evidence of NK cell adaptation to HCMV infection does not equate with superior CD16-mediated effector functions. The finding here most consistent with previous studies using donors not infected with HIV was the correlation between CD16-triggered IFN-γ production and loss of FcRγ adaptor subunits (25, 26). Whether loss of FcRγ is a marker for other alterations or its absence plays a direct role in enhanced signaling through CD16, this aspect of NK cell adaptation to HCMV infection appears to be maintained in HIV infection. We saw no relationship between FcRγ expression and either ADCC measured by ^51^Cr release or CD16-triggered degranulation, lending credence to speculation that NK cell adaptation to HCMV affects CD16-mediated cytokine production more so than it affects cytotoxicity.

To investigate NK cell progression toward terminal differentiation, we assessed PD-1, Lag-3 and Tim-3 expression. We detected little to no PD-1 or Lag-3 in any of the three groups, yet a significant fraction of NK cells expressed Tim-3. Tim-3 expression levels did not differ between groups and were unaffected by CD16 stimulation. Although recent reports suggest enhanced function of Tim-3^pos^ NK cells, we observed functional deficits in degranulation and IFN-γ production in the HCMV^neg^ and HCMV^pos^ groups, respectively, relative to the general NK cell population (46). Although significant, these deficits were relatively slight and based on the responses of a small group of HIV-infected individuals. Further study of the role Tim-3 has on NK cell functions in different settings is warranted.

In summary, type I IFNs produced during *in vitro* HCMV infection of fibroblasts increased NK cell cytotoxicity through multiple receptors. This increase in NK cytotoxicity occurred with NK cells from HCMV-seronegative and seropositive healthy controls. In the HIV-infected subjects, we saw a similar increase in ADCC that was unrelated to HCMV status or extent of NK cell adaptation to HCMV infection. Cytokine and degranulation responses mediated through CD16 were well preserved in the HIV-infected individuals we studied, again unrelated to their HCMV status. Despite an inverse correlation overall between NK cell CD16-triggered IFN-γ production and FcRγ expression, the HCMV^neg^, HIV-infected subject group did not have a significantly lesser IFN-γ response than either of the groups with higher fractions of FcRγ^neg^ NK cells. There was no evidence that NK cell adaptation to HCMV affects degranulation responses or cytotoxicity triggered through CD16 in HIV infection. While these findings suggest that HCMV-related NK cell adaptation has different or lesser functional consequence in HIV infection, the clear effect of HCMV on FcRγ expression and the extreme levels of adaptation observed in terms of NKG2C expression illustrate the same selectivity operating with increased pressure. Immunological pressures associated with HIV infection may preserve or enhance NK cell function through compensatory pathways distinct from HCMV-driven adaptation.

## Author contributions

The study was conceived and planned by MG and KH. Experimental work, data analysis, and data presentation was primarily performed by KH. JL carried out the luminex analysis on supernatant from CON, HCMV, and vSC8-infected cells. The manuscript was drafted and revised by KH and MG.

### Conflict of interest statement

The authors declare that the research was conducted in the absence of any commercial or financial relationships that could be construed as a potential conflict of interest.

## References

[1] Kim S Poursine-Laurent J Truscott SM Lybarger L Song YJ Yang L. Licensing of natural killer cells by host major histocompatibility complex class I molecules. Nature (2005) 436:709–13. 10.1038/nature03847 16079848

[2] Raulet DH Vance RE. Self-tolerance of natural killer cells. Nat Rev Immunol. (2006) 6:520–31. 10.1038/nri1863 16799471

[3] Yokoyama WM Kim S. Licensing of natural killer cells by self-major histocompatibility complex class. Immunol Rev I. (2006) 214:143–54. 10.1111/j.1600-065X.2006.00458.x 17100882

[4] Brodin P Hoglund P. Beyond licensing and disarming: a quantitative view on NK-cell education. Eur J Immunol. (2008) 38:2934–7. 10.1002/eji.200838760 18979511

[5] Jonsson AH Yokoyama WM. Natural killer cell tolerance licensing and other mechanisms. Adv Immunol. (2009) 101:27–79. 10.1016/S0065-2776(08)01002-X 19231592

[6] Brown MG Dokun AO Heusel JW Smith HR Beckman DL Blattenberger EA. Vital involvement of a natural killer cell activation receptor in resistance to viral infection. Science (2001) 292:934–7. 10.1126/science.1060042 11340207

[7] Daniels KA Devora G Lai WC O'Donnell CL Bennett M Welsh RM. Murine cytomegalovirus is regulated by a discrete subset of natural killer cells reactive with monoclonal antibody to Ly49H. J Exp Med. (2001) 194:29–44. 10.1084/jem.194.1.29 11435470 PMC2193438

[8] Smith HR Heusel JW Mehta IK Kim S Dorner BG Naidenko OV. Recognition of a virus-encoded ligand by a natural killer cell activation receptor. Proc Natl Acad Sci USA. (2002) 99:8826–31. 10.1073/pnas.092258599 12060703 PMC124383

[9] Arase H Mocarski ES Campbell AE Hill AB Lanier LL. Direct recognition of cytomegalovirus by activating and inhibitory NK cell receptors. Science (2002) 296:1323–6. 10.1126/science.1070884 11950999

[10] Vidal SM Lanier LL. NK cell recognition of mouse cytomegalovirus-infected cells. Curr Top Microbiol Immunol. (2006) 298:183–206. 16329187 10.1007/3-540-27743-9_10

[11] Sun JC Beilke JN Lanier LL. Adaptive immune features of natural killer cells. Nature (2009) 457:557–61. 10.1038/nature07665 19136945 PMC2674434

[12] Madera S Sun JC. Cutting edge: stage-specific requirement of IL-18 for antiviral NK cell expansion. J Immunol. (2015) 194:1408–12. 10.4049/jimmunol.1402001 25589075 PMC4323636

[13] Sun JC Madera S Bezman NA Beilke JN Kaplan MH Lanier LL. Proinflammatory cytokine signaling required for the generation of natural killer cell memory. J Exp Med. (2012) 209:947–54. 10.1084/jem.20111760 22493516 PMC3348098

[14] Cooper MA Elliott JM Keyel PA Yang L Carrero JA Yokoyama WM. Cytokine-induced memory-like natural killer cells. Proc Natl Acad Sci USA. (2009) 106:1915–9. 10.1073/pnas.0813192106 19181844 PMC2644138

[15] Tripathy SK Smith HR Holroyd EA Pingel JT Yokoyama WM. Expression of m157, a murine cytomegalovirus-encoded putative major histocompatibility class I (MHC-I)-like protein, is independent of viral regulation of host MHC-I. J Virol. (2006) 80:545–50. 10.1128/JVI.80.1.545-550.2006 16352579 PMC1317552

[16] Bubic I Wagner M Krmpotic A Saulig T Kim S Yokoyama WM. Gain of virulence caused by loss of a gene in murine cytomegalovirus. J Virol. (2004) 78:7536–44. 10.1128/JVI.78.14.7536-7544.2004 15220428 PMC434107

[17] Voigt V Forbes CA Tonkin JN Degli-Esposti MA Smith HR Yokoyama WM. Murine cytomegalovirus m157 mutation and variation leads to immune evasion of natural killer cells. Proc Natl Acad Sci USA. (2003) 100:13483–8. 10.1073/pnas.2233572100 14597723 PMC263840

[18] Guma M Angulo A Vilches C Gomez-Lozano N Malats N Lopez-Botet M. Imprint of human cytomegalovirus infection on the NK cell receptor repertoire. Blood (2004) 104:3664–71. 10.1182/blood-2004-05-2058 15304389

[19] Lopez-Verges S Milush JM Pandey S York VA Arakawa-Hoyt J Pircher H. CD57 defines a functionally distinct population of mature NK cells in the human CD56dimCD16+ NK-cell subset. Blood (2010) 116:3865–74. 10.1182/blood-2010-04-282301 20733159 PMC2981540

[20] Lopez-Verges S Milush JM Schwartz BS Pando MJ Jarjoura J York VA. Expansion of a unique CD57(+)NKG2Chi natural killer cell subset during acute human cytomegalovirus infection. Proc Natl Acad Sci USA. (2011) 108:14725–32. 10.1073/pnas.1110900108 21825173 PMC3169160

[21] Foley B Cooley S Verneris MR Curtsinger J Luo X Waller EK. Human cytomegalovirus (CMV)-induced memory-like NKG2C(+) NK cells are transplantable and expand *in vivo* in response to recipient CMV antigen. J Immunol. (2012) 189:5082–8. 10.4049/jimmunol.1201964 23077239 PMC3490031

[22] Guma M Budt M Saez A Brckalo T Hengel H Angulo A. Expansion of CD94/NKG2C+ NK cells in response to human cytomegalovirus-infected fibroblasts. Blood (2006) 107:3624–31. 10.1182/blood-2005-09-3682 16384928

[23] Rolle A Pollmann J Ewen EM Le VT Halenius A Hengel H. IL-12-producing monocytes and HLA-E control HCMV-driven NKG2C+ NK cell expansion. J Clin Invest. (2014) 124:5305–16. 10.1172/JCI77440 25384219 PMC4348979

[24] Hammer Q Ruckert T Borst EM Dunst J Haubner A Durek P. Peptide-specific recognition of human cytomegalovirus strains controls adaptive natural killer cells. Nat Immunol. (2018) 19:453–463. 10.1038/s41590-018-0082-6 29632329

[25] Hwang I Zhang T Scott JM Kim AR Lee T Kakarla T. Identification of human NK cells that are deficient for signaling adaptor FcRgamma and specialized for antibody-dependent immune functions. Int Immunol. (2012) 24:793–802. 10.1093/intimm/dxs080 22962434 PMC3621379

[26] Zhang T Scott JM Hwang I Kim S. Cutting edge: antibody-dependent memory-like NK cells distinguished by FcRgamma deficiency. J Immunol. (2013) 190:1402–6. 10.4049/jimmunol.1203034 23345329 PMC3623944

[27] Wu Z Sinzger C Frascaroli G Reichel J Bayer C Wang L. Human cytomegalovirus-induced NKG2C(hi) CD57(hi) natural killer cells are effectors dependent on humoral antiviral immunity. J Virol. (2013) 87:7717–25. 10.1128/JVI.01096-13 23637420 PMC3700275

[28] Schlums H Cichocki F Tesi B Theorell J Beziat V Holmes TD. Cytomegalovirus infection drives adaptive epigenetic diversification of NK cells with altered signaling and effector function. Immunity (2015) 42:443–56. 10.1016/j.immuni.2015.02.008 25786176 PMC4612277

[29] Holder KA Comeau EM Grant MD. Origins of natural killer cell memory: special creation or adaptive evolution. Immunology (2018) 154:38–49. 10.1111/imm.12898 29355919 PMC5904722

[30] Campos C Pera A Sanchez-Correa B Alonso C Lopez-Fernandez I Morgado S. Effect of age and CMV on NK cell subpopulations. Exp Gerontol. (2014) 54:130–7. 10.1016/j.exger.2014.01.008 24440462

[31] Kuijpers TW Baars PA Dantin C van den Burg M van Lier RA Roosnek E. Human NK cells can control CMV infection in the absence of T cells. Blood (2008) 112:914–5. 18650467 10.1182/blood-2008-05-157354

[32] Foley B Cooley S Verneris MR Pitt M Curtsinger J Luo X. Cytomegalovirus reactivation after allogeneic transplantation promotes a lasting increase in educated NKG2C+ natural killer cells with potent function. Blood (2012) 119:2665–74. 10.1182/blood-2011-10-386995 22180440 PMC3311280

[33] Hadaya K de Rham C Bandelier C Bandelier C Ferrari-Lacraz S Jendly S. Natural killer cell receptor repertoire and their ligands, and the risk of CMV infection after kidney transplantation. Am J Transplant. (2008) 8:2674–83. 10.1111/j.1600-6143.2008.02431.x 19032228

[34] Heath J Newhook N Comeau E Gallant M Fudge N Grant M. NKG2C(+)CD57(+) natural killer cell expansion parallels cytomegalovirus-specific CD8(+) T cell evolution towards senescence. J Immunol Res. (2016) 2016:7470124. 10.1155/2016/7470124 27314055 PMC4903150

[35] Barrett L Fudge NJ Heath JJ Grant MD. Cytomegalovirus immunity and exhaustive CD8+ T cell proliferation in treated human immunodeficiency virus infection. Clin Infect Dis. (2016) 62:1467–8. 10.1093/cid/ciw148 27001798

[36] Rowe WP Huebner RJ Gilmore LK Parrott RH Ward TG. Isolation of a cytopathogenic agent from human adenoids undergoing spontaneous degeneration in tissue culture. Proc Soc Exp Biol Med. (1953) 84:570–3. 10.3181/00379727-84-20714 13134217

[37] Paust S Blish CA Reeves RK. Redefining memory: building the case for adaptive NK cells. J Virol. (2017) 91:e00169–17. 10.1128/JVI.00169-17 28794018 PMC5625515

[38] Lajoie J Juno J Burgener A Rahman S Mogk K Wachihi C. A distinct cytokine and chemokine profile at the genital mucosa is associated with HIV-1 protection among HIV-exposed seronegative commercial sex workers. Mucosal Immunol. (2012) 5:277–87. 10.1038/mi.2012.7 22318497

[39] Luetke-Eversloh M Cicek BB Siracusa F Thom JT Hamann A Frischbutter S. NK cells gain higher IFN-gamma competence during terminal differentiation. Eur J Immunol. (2014) 44:2074–84. 10.1002/eji.201344072 24752800

[40] Luetke-Eversloh M Hammer Q Durek P Nordstrom K Gasparoni G Pink M. Human cytomegalovirus drives epigenetic imprinting of the IFNG locus in NKG2Chi natural killer cells. PLoS Pathog. (2014) 10:e1004441. 10.1371/journal.ppat.1004441 25329659 PMC4199780

[41] Bjorkstrom NK Lindgren T Stoltz M Fauriat C Braun M Evander M. Rapid expansion and long-term persistence of elevated NK cell numbers in humans infected with hantavirus. J Exp Med. (2011) 208:13–21. 10.1084/jem.20100762 21173105 PMC3023129

[42] Petitdemange C Becquart P Wauquier N Beziat V Debre P Leroy EM. Unconventional repertoire profile is imprinted during acute chikungunya infection for natural killer cells polarization toward cytotoxicity. PLoS Pathog. (2011) 7:e1002268. 10.1371/journal.ppat.1002268 21966274 PMC3178577

[43] Della Chiesa M Falco M Bertaina A Muccio L Alicata C Frassoni F. Human cytomegalovirus infection promotes rapid maturation of NK cells expressing activating killer Ig–like receptor in patients transplanted with NKG2C-/- umbilical cord blood. J Immunol. (2014) 192:1471–9. 10.4049/jimmunol.1302053 24442432

[44] Goodier MR White MJ Darboe A Nielsen CM Goncalves A Bottomley C. Rapid NK cell differentiation in a population with near-universal human cytomegalovirus infection is attenuated by NKG2C deletions. Blood (2014) 124:2213–22. 10.1182/blood-2014-05-576124 25150297 PMC4206953

[45] Liu LL Landskron J Ask EH Enqvist M Sohlberg E Traherne JA. Critical role of CD2 co-stimulation in adaptive natural killer cell responses revealed in NKG2C-deficient humans. Cell Rep. (2016) 15:1088–99. 10.1016/j.celrep.2016.04.005 27117418 PMC4858565

[46] Kared H Martelli S Tan SW Simoni Y Chong ML Yap SH. Adaptive NKG2C(+)CD57(+) natural killer cell and Tim-3 expression during viral infections. Front Immunol. (2018) 9:686. 10.3389/fimmu.2018.00686 29731749 PMC5919961

